# Biological Reference Materials for Trace Element Analysis: What is New?

**DOI:** 10.6028/jres.093.060

**Published:** 1988-06-01

**Authors:** J. Versieck, L. Vanballenberghe, A. De Kesel, J. Hoste, B. Wallaeys, J. Vandenhaute

**Affiliations:** Department of Internal Medicine, Division of Gastroenterology, University Hospital, De Pintelaan 185, B-9000 Ghent; Laboratory for Analytical Chemistry, Institute for Nuclear Sciences, Proeftuinstraat 86, B-9000 Ghent, Belgium

## Introduction

In biomedical trace element research, reference materials are a relatively recent development: indeed, Bowen’s Kale Powder became available only in the middle of the 1960s, NBS Orchard Leaves (SRM 1571) and NBS Bovine Liver (SRM 1577) in the beginning of the 1970s. In general, when compared to a number of real samples submitted to laboratories for elemental analyses, these reference materials have high or relatively high trace element concentrations. This is probably best illustrated when the levels expected in human blood plasma or serum (matrices which are frequently analyzed both because of their established biomedical importance and their ready availability) are considered. For example, for chromium and manganese, figures are as follows—370 and 14,950 ng/g (in Bowen’s Kale Powder), 88 and 10,300 ng/g (in NBS Bovine Liver), and 1.5 and 5.5 ng/g dry weight (in human blood plasma or serum). So, although the above-mentioned, first generation biological reference materials rendered excellent services as a means to check the accuracy and precision of analytical techniques, large numbers of investigators were still largely left to their own devices.

Some reference materials are specifically aimed at researchers working on human blood plasma or serum, e.g., NBS Human Serum (SRM 909), Nycomed’s Human Serum (STE 105), and National Institute for Environmental Studies’ (NIES) (Japan) Freeze-Dried Human Serum. In all three cases, however, it is obvious that the original levels of several elements must have been drastically distorted during the collection and preparation of the materials. For example, in reconstituted NBS Human Serum the nominal concentration of chromium is 91.3 ng/mL whereas the true level of the element in fresh plasma or serum is now widely believed to be approximately 0.15 ng/mL (or approximately 1.5 ng/g dry weight as mentioned above); in reconstituted Nycomed’s Human Serum, the concentration of manganese was measured to be 23.5 ng/mL whereas the true level of the element in plasma or serum is now definitively established to be approximately 0.55 ng/mL (or approximately 5.5 ng/g dry weight as mentioned above). In fact, these materials give false feelings of confidence to inexperienced workers in search of reference materials to back their figures in real plasma or serum samples; obviously, they are only useful in a few instances, namely for the determination of iron, copper, and zinc, and, in the case of Nycomed’s Human Serum, for the determination of selenium.

## New Developments

Among researchers working on matrices with low trace element concentrations (or with “ultratrace” element concentrations), the need for second generation biological reference materials with trace element levels close to those in real samples was very strongly felt. Efforts to meet their needs were undertaken in some centers.

## NBS Non-Fat Milk Powder, SRM 1549

This material, issued in 1984, represents a matrix with some of the lowest trace element levels so far available. To the authors of this paper, not much is known about its collection and preparation. Recent publications [1,2] give very few details. Trace element levels (either certified or information values) are listed in table 1. Several years before (in the late 1970s), the International Atomic Energy Agency (IAEA) also issued a milk powder reference material (A-11). However, in the original intercomparisons it gave very unsatisfactory results for most elements so that a coordinated program was set up to improve the certification: the results were published only some months ago [3]. The most striking differences are a markedly higher chromium (17.7 ng/g) and mercury level (3.2 ng/g), a moderately higher arsenic (4.85 ng/g) and cadmium level (1.7 ng/g), but a definitely lower molybdenum level (92 ng/g) in the IAEA material.

## NBS Bovine Serum, RM 8419

This is a very important material that became available in 1985 (pool of approximately 8 liters). Its collection and preparation (by C. Veillon and coworkers in Beltsville, MD) as well as its characterization (by C. Veillon and collaborating investigators both from Europe and the United States) were described [4,5]. It is interesting to note that the material was distributed in liquid form and that its recommended values were in *μ*g/L or mg/L (ng/ml or *μ*g/ml); for the sake of uniformity, however, values listed in table 1 were converted into ng/g or *μ*g/g dry weight. Its main characteristics are obvious from the table. This material has now been analyzed by several researchers throughout the world. As indicated, in a footnote to the table, for nickel, values lower than that assigned were obtained by Andersen et al. [6]. As far as the authors of this paper are aware, the first batch of this material is now exhausted but a second is in preparation; preliminary results for a number of elements are similar to those measured in the first batch.

## Freeze-Dried Human Serum

This second generation reference material was developed at the University of Ghent in Belgium by the authors. In 1981, a pilot study was set up to test the feasibility of the project; collections for the final pool were started in the second half of 1982. As donors, patients were selected for idiopathic hemochromatosis under treatment with phlebotomy. All were tested for hepatitis B surface antigen (HBsAg) as well as for antibodies against human immunodeficiency virus (HIV) and found to be negative. In December 1984, approximately 22 liters were accumulated. The collection and preparation of the material were discussed at the 7th International Conference on Modern Trends in Activation Analysis (Copenhagen, 1986); the written version of the text is in press [7]. The most important point that should be emphasized is that all imaginable precautions were observed to avoid contamination with exogeneous material! In 1985 and 1986, the material was homogenized and certified. A description of the whole process has been submitted for publication [8]. The values attached to the material are also catalogued in table 1. A comparison of the levels with those expected in lyophilized plasma or serum samples of apparrently healthy adults illustrates that the purpose of the project—preparing a reference material with low trace element levels, comparable to those in one of the most challenging matrices: real plasma or serum—was attained. Particular attention is drawn to the values for chromium, manganese, nickel, molybdenum, cadmium, and other difficult elements to measure reliably.

## Conclusion

During the last 5 years, second generation biological reference materials have became available. Investigators engaged in low level trace element research finally have the means to check the accuracy and precision of their analytical procedures in the best possible conditions; in addition, for the first time they have at their disposal a possibility to evaluate the adequacy of some of their sample manipulation and preparation methods. With judicious use of the newly developed reference materials, it should be possible within a relatively short period of time to harmonize low-level trace element measurements by different researchers in human health and disease.

## Figures and Tables

**Table 1:** Trace element concentrations in recently developed biological reference materials

Element, unit	NBS Non-Fat Milk Power, SRM 1549	NBS Bovine Serum, RM 8419^a^	Freeze-Dried Human Serum, this paper
1Al, ng/g dry weight	(2000)	132	20.2
Cr, ng/g	2.6	3.04	0.76
Mn, ng/g	260	26.4	7.7
Fe, *μ*g/g	(2.1)	20.3	25.9
Co, ng/g	(4.1)	12.2	3.6
Ni, ng/g		18.3^b^	(2.5)
Cu, *μ*g/g	0.7	7.6	11.1
Zn, *μ*g/g	46.1	11.2	9.6
As, ng/g	(1.9)	(3.5)	19.6
Se, *μ*g/g	0.11	0.16	1.05
Br, *μ*g/g	(12)		48.8
Rb, *μ*g/g	(11)	(1.20)	1.85
Mo, ng/g	(340)	162	7.5
Cd, ng/g	0.5		2.0
Cs, ng/g		(2.2)	10.0
Hg, ng/g	0.3		(7.0)

a Values converted into ng/g or μg/g dry weight: recommended values, published in μg/L or mg/L [5], were multiplied by 10.15 (i.e., 100/(1.026×9.6)−1.026 being the specific gravity of the fresh serum (estimated) and 9.6 the percent residue after freeze-drying).

b Recently, lower values were reported by other researchers [6].

Figures in parentheses are information values only; the others are certified (NBS Non-Fat Milk Powder, SRM 1549, and Freeze-Dried Human Serum, this paper) or recommended values (NBS Bovine Serum, RM 8419).
